# Obesity’s systemic impact: exploring molecular and physiological links to diabetes, cardiovascular disease, and heart failure

**DOI:** 10.3389/fendo.2025.1681766

**Published:** 2025-11-07

**Authors:** Dipanjan Banerjee, Arya Mani

**Affiliations:** 1 Cardiovascular Research Center, Department of Internal Medicine, Yale University School of Medicine, New Haven, CT, United States; 2 Department of Genetics, Yale University School of Medicine, New Haven, CT, United States

**Keywords:** obesity, inflammation, insulin resistance, atherosclerosis, heart failure

## Abstract

Obesity prevalence continues to climb globally, driving healthcare costs ever higher. Over the past decade, significant strides have been made in understanding the causes of obesity, revealing that primary obesity is rooted in a complex interplay of genetic/developmental and epigenetic/environmental factors. Despite this progress, a critical gap remains in our understanding of the precise molecular pathways that translate adipose tissue expansion into the vast spectrum of associated comorbidities and heterogeneous patient outcomes. This review aims to synthesize recent mechanistic insights that bridge this gap. We summarize findings from extensive literature searches to highlight recent discoveries in the mechanisms underlying obesity and elucidate how these mechanisms contribute to various comorbidities. This review explores key pathways, including inflammation, insulin resistance, adipokine dysregulation, and complement system activation, that link obesity to diabetes, cardiovascular diseases, and metabolic syndrome. We provide a focused analysis of how these pathways drive two major obesity-related conditions: type 2 diabetes and cardiovascular disease, with particular emphasis on the pathophysiological mechanisms leading to heart failure. Additionally, we discuss the pathophysiological changes induced by obesity that directly contribute to the development of heart failure, including alterations in cardiac structure and function. Our findings highlight the intricate relationships between obesity and its comorbidities, emphasizing the need for a deeper understanding of these mechanisms to inform targeted interventions, druggable pathways, and improve management strategies for affected individuals.

## 1 Introduction

Obesity prevalence continues to climb globally, posing an unprecedented public health challenge and significantly escalating healthcare costs. It is now recognized as one of the leading preventable causes of morbidity and mortality worldwide. Since the 1970s, the global rate of obesity has nearly tripled, making it an epidemic of the 21st century. In 2016 alone, over 1.9 billion adults—equating to 39% of the global adult population—were overweight, with more than 650 million categorized as obese (1). Alarmingly, this trend is not confined to adults; over 41 million children under the age of five and 340 million children and adolescents aged 5–19 were overweight or obese, pointing to early-life origins of metabolic disease (1). In the United States, the situation is particularly dire, where 40% of adults and about 20% of children and adolescents (ages 6–19) met criteria for obesity during the 2015–2016 reporting cycle (2, 3).

Recent epidemiological studies confirm that obesity contributes to approximately 2.8–3.4 million deaths annually, with growing recognition that it serves as a primary driver for a constellation of chronic diseases—including cardiovascular disease (CVD), type 2 diabetes mellitus (T2DM), non-alcoholic fatty liver disease (NAFLD), and multiple types of cancer (2, 3). The pathophysiological basis of these associations has become increasingly clear over the past decade, highlighting obesity not merely as an excess of adipose tissue but as a chronic low-grade inflammatory and endocrine disease that disrupts systemic homeostasis.

**Figure 1:**
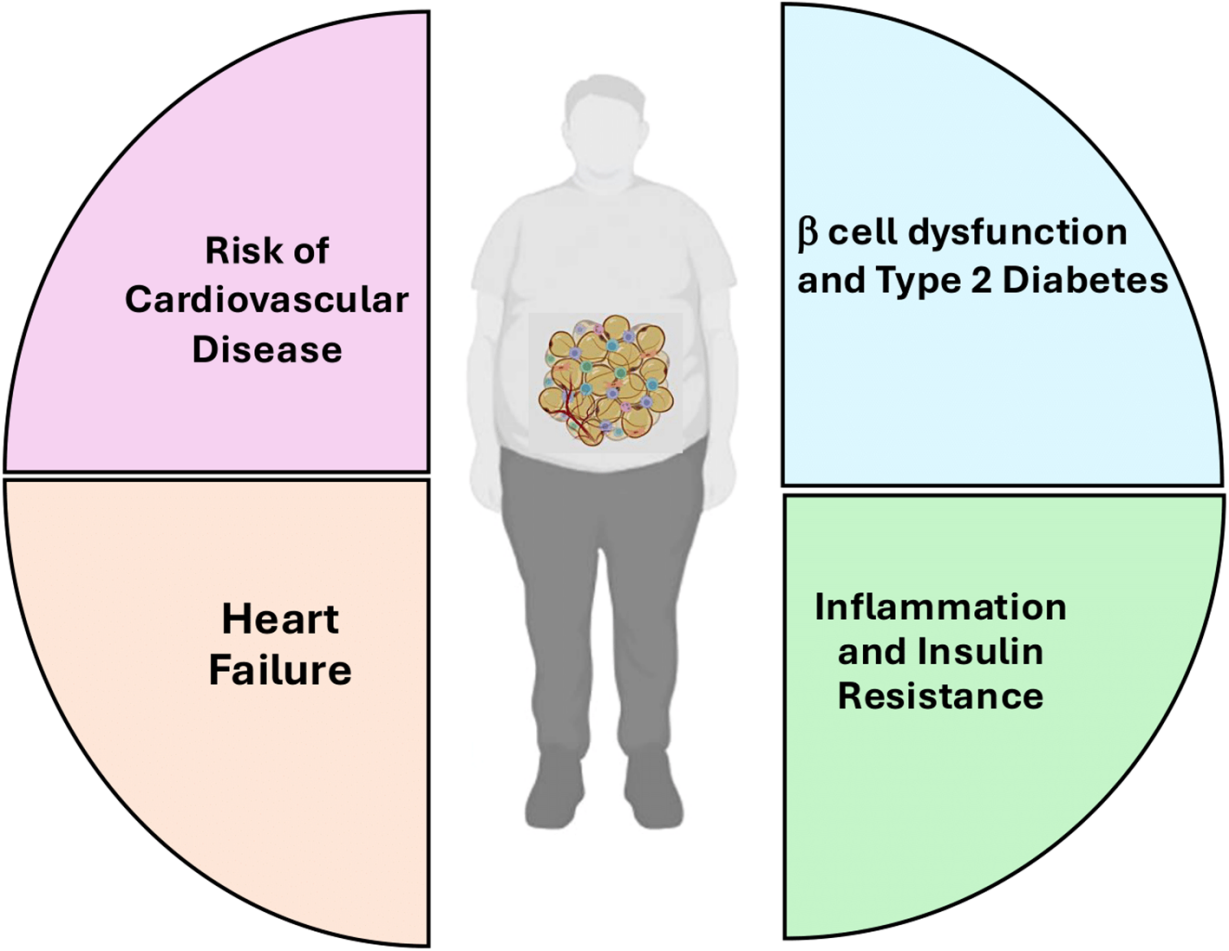
The multifaceted impact of obesity on health. This diagram illustrates how obesity contributes to several major health complications. These include an increased risk of cardiovascular disease, the development of heart failure, β-cell dysfunction leading to Type 2 Diabetes, and systemic inflammation and insulin resistance.

Obesity is characterized by a pathological expansion of adipose tissue through both hypertrophy (increase in adipocyte size) and hyperplasia (increase in adipocyte number) (4, 5). This expansion is not metabolically inert: adipocytes secrete a range of adipokines, cytokines, and chemokines that exert local and systemic effects on metabolism, immunity, and organ function. Obese individuals—especially those with visceral fat accumulation—are significantly more likely to develop comorbid conditions that cluster into a high-risk phenotype known as *cardiometabolic multimorbidity* (6, 7) (**Figure 1**). A major multicohort study of over 120,000 adults found that individuals with even mild obesity (BMI 30–34.9 kg/m²) were over four times more likely to exhibit two or more of the following conditions: myocardial infarction, stroke, and T2DM (8, 9). Similarly, another study of more than 11,000 participants demonstrated that overweight and obese individuals had a twofold increased risk of developing hypertension, dyslipidemia, atherosclerosis, and cardiomyopathy (10, 11).

Despite decades of epidemiological and mechanistic research, investigations have only begun to delineate the molecular pathways by which obesity triggers and accelerates these comorbidities. A critical knowledge gap remains in understanding how obesity—beyond its definition as excess adiposity—translates into heterogeneous health outcomes. While visceral fat accumulation, chronic inflammation, and adipokine dysregulation are recognized as central drivers of cardiometabolic disease, the precise mechanisms linking adipose tissue expansion to organ-specific pathologies, multimorbidity, and differential risk profiles remain incompletely defined. Furthermore, paradoxical observations—such as protective effects of subcutaneous fat or the “obesity paradox” in certain populations—highlight unresolved questions about fat distribution, metabolic adaptability, and individual variability in disease progression. Addressing this gap is essential to move beyond BMI-centric definitions and toward mechanistic insights that can inform precision prevention and targeted therapies.

In this review, we synthesize recent findings to elucidate the biological mechanisms by which obesity contributes to a wide spectrum of systemic pathologies. A central feature of obesity is chronic, low-grade inflammation, which not only promotes insulin resistance but also drives endothelial dysfunction, thereby linking excess adiposity to both metabolic and cardiovascular complications. Another critical pathway is adipokine dysregulation. Obesity alters the secretion of adipose-derived hormones such as leptin, adiponectin, and resistin, and these imbalances have profound effects on metabolic homeostasis and cardiovascular health. Insulin resistance represents a further hallmark of obesity, disrupting both glucose and lipid metabolism and forming the core defect in the pathogenesis of type 2 diabetes mellitus. Beyond these established mechanisms, complement system activation has recently emerged as an additional contributor, with growing evidence implicating complement components in adipose tissue inflammation, metabolic dysregulation, and lipid handling. Together, these interlinked pathways highlight the multifactorial nature of obesity-associated disease and underscore the importance of integrating emerging insights with established paradigms.

We also explore how these mechanisms converge to cause structural and functional changes in the cardiovascular system—ranging from endothelial injury and atherosclerotic plaque development to cardiac fibrosis and heart failure. Furthermore, we evaluate how obesity may disrupt organ crosstalk (e.g., between adipose tissue, the liver, and pancreas), giving rise to systemic metabolic derangements such as dyslipidemia, hepatic steatosis, and β-cell dysfunction.

A particular emphasis is placed on the role of the *innate immune system*, including macrophage polarization, toll-like receptor (TLR) signaling, and the activation of complement factors such as C3a, C3adesArg (also known as acylation-stimulating protein, ASP), and C5a—all of which have emerged as critical modulators of adipose tissue inflammation, insulin sensitivity, and cardiovascular health.

Together, these insights illuminate how obesity-induced perturbations in immune, endocrine, and metabolic pathways culminate in a state of *multisystem dysfunction*. Our aim is to provide an integrative and up-to-date overview of the mechanisms linking obesity to comorbid diseases and to highlight how this knowledge may inform the development of *targeted pharmacological and lifestyle-based interventions*. In doing so, we also advocate for a paradigm shift—moving from viewing obesity as an isolated condition to recognizing it as a *root cause of complex chronic multimorbidity*.

## 2 Inflammation and insulin resistance promote T2DM

Obesity arises when caloric intake persistently exceeds energy expenditure, resulting in excess energy being stored as triglycerides within adipose tissue. This positive energy balance is influenced by a constellation of factors—environmental (e.g., sedentary lifestyle), neurological (e.g., hypothalamic regulation of appetite), genetic predispositions, and hormonal or metabolic imbalances. The resultant expansion of adipose tissue leads to profound systemic metabolic disturbances, including chronic inflammation, oxidative stress, and dyslipidemia. These early hallmarks of obesity—characterized by elevated circulating triacylglycerols (TAG), increased low-density lipoprotein (LDL), reduced high-density lipoprotein (HDL), and insulin resistance—constitute the metabolic syndrome (12, 13). If left unchecked, these perturbations set the stage for the progression to type 2 diabetes mellitus (T2DM), cardiovascular disease, and premature death, particularly in genetically or environmentally susceptible individuals.

Obesity more than doubles the risk of developing metabolic syndrome and increases the risk of T2DM by fourfold (14). T2DM—accounting for over 90% of all diabetes cases—is driven by a dual pathology: *peripheral insulin resistance and pancreatic β-cell dysfunction*, both of which are profoundly influenced by chronic inflammation. In obesity, the ability of peripheral tissues (e.g., skeletal muscle, liver, adipose) to respond to insulin is impaired, increasing the insulin demand on β-cells. Initially, β-cells compensate by enhancing insulin secretion, but over time, they become dysfunctional, fail to meet metabolic demands, and undergo apoptosis—culminating in overt hyperglycemia and diabetes.

### 2.1 β-cell physiology in an obese condition

Obesity is associated with increased fat infiltration in multiple organs, including the pancreas. As ectopic lipid accumulation intensifies within islets, pancreatic β-cells are exposed to lipotoxic stress. In rodent models, this leads to β-cell dysfunction, dedifferentiation, and apoptosis (15, 16). Paradoxically, in the early stages of obesity, β-cells adapt by increasing their mass in response to insulin resistance—a process that occurs via β-cell replication and neogenesis from progenitors.

Experimental studies in rats have shown that high-fat diet (HFD)-induced obesity can cause a threefold increase in β-cell mass, mainly due to enhanced proliferation (17). Similar findings were reported in mouse models, where diet-induced obesity (DIO) leads to an increase in β-cell area and insulin-positive cells. In human autopsy studies, obese individuals without diabetes often show a 20–90% increase in β-cell mass compared to lean individuals (18). However, the exact timing, extent, and sustainability of this compensation vary between individuals and are influenced by age, duration of obesity, and genetic factors.

### 2.2 β-cell dysfunction and the onset of T2DM

Although β-cell compensation initially delays the onset of hyperglycemia, progressive β-cell failure marks the transition from insulin resistance to overt T2DM. In individuals with diabetes, β-cell loss is attributed to several mechanisms:

- Apoptosis, which is elevated in human T2DM islets (15);
- Reduced proliferation or regenerative capacity, which is limited in adult humans (19).
- Oxidative stress and endoplasmic reticulum (ER) stress, which impair protein folding and insulin biosynthesis (18);
- Inflammatory cytokines (e.g., IL-1β, TNF-α) produced locally or systemically, which activate β-cell death pathways (20);
- Lipotoxicity, where prolonged exposure to free fatty acids, especially saturated fats, impairs insulin gene expression and mitochondrial function (21);
- Glucotoxicity, where chronic hyperglycemia induces β-cell oxidative damage (22);
- Amyloid deposition, which disrupts β-cell membranes (23);
- Autophagy defects, reducing the clearance of damaged organelles (24).

**Figure 2:**
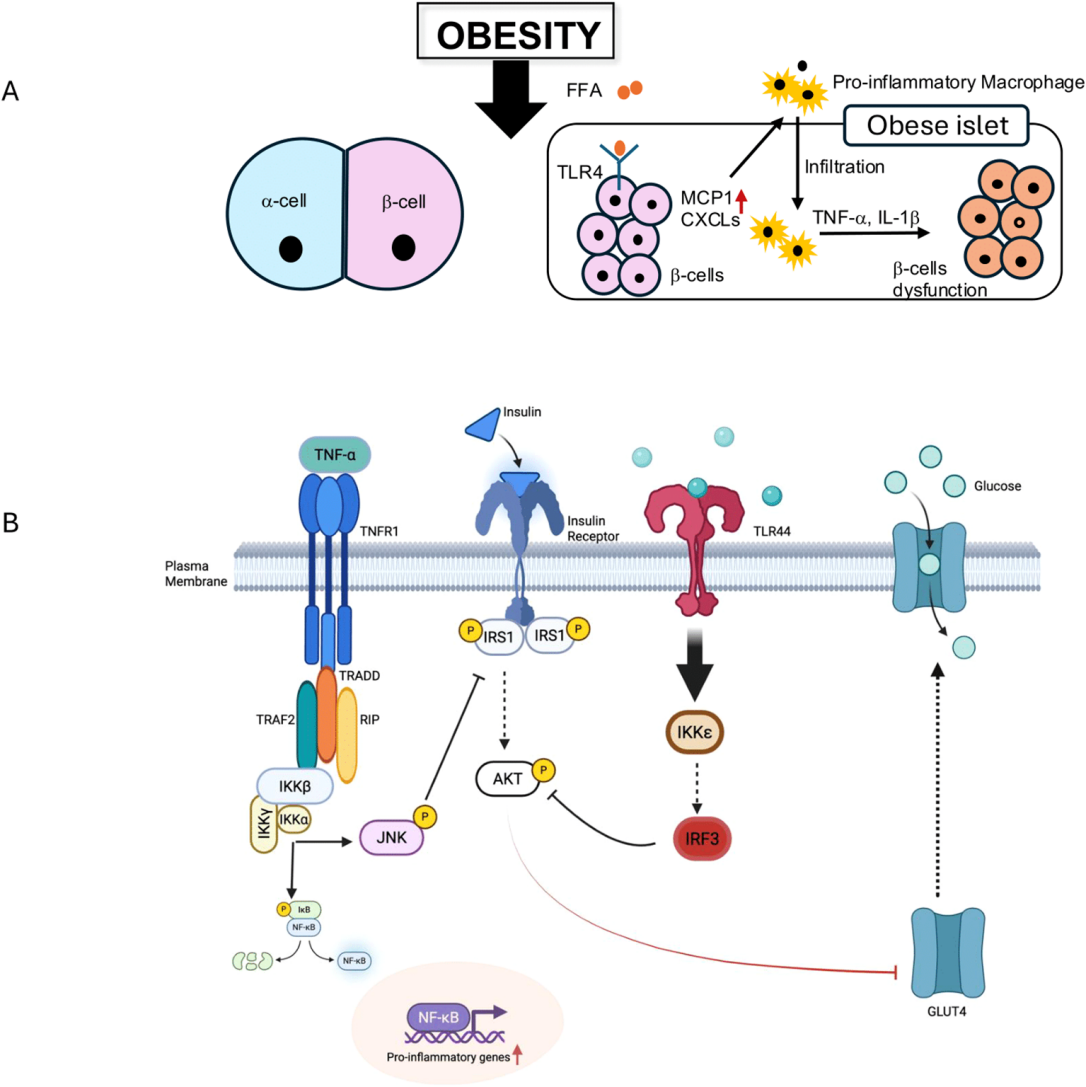
Mechanisms of obesity-induced β-cell dysfunction and insulin resistance. **(A)** Obesity leads to increased free fatty acids (FFAs), which activate TLR4 on pancreatic β-cells. This activation, along with other signals, promotes the recruitment of pro-inflammatory macrophages and the production of pro-inflammatory cytokines like TNF-α and IL-1β, leading to infiltration of immune cells into the islet and resulting in β-cell dysfunction. **(B)** A schematic illustrating molecular pathways contributing to insulin resistance and inflammation. TNF-α binds to TNFR1, activating a signaling cascade involving TRADD, TRAF2, RIP, IKKβ, and JNK. IKKβ activation leads to NF-κB activation and the transcription of pro-inflammatory genes. JNK activation phosphorylates IRS1 at Serine residue, inhibiting its ability to signal downstream through AKT, thereby impairing insulin signaling and glucose uptake via GLUT4. Additionally, TLR4 activation by FFAs can directly activate IKKε and IRF3, further contributing to inflammatory responses and insulin resistance.

An emerging mechanism of β-cell loss involves *transdifferentiation*—where β-cells lose their identity or convert into other cell types, such as α-cells (**Figure 2A**). Rodent studies have confirmed this phenomenon under stress conditions (25), and human studies suggest that dedifferentiated β-cells—marked by the loss of insulin and gain of progenitor-like markers—are more frequent in T2DM islets (26).

### 2.3 Adipose tissue biology and insulin resistance

Adipose tissue is a dynamic endocrine organ that secretes adipokines, cytokines, and extracellular vesicles to regulate systemic metabolism, energy homeostasis, and immune responses. In lean states, adipocytes maintain insulin sensitivity and release anti-inflammatory adipokines such as adiponectin. However, during chronic overnutrition, adipocytes undergo hypertrophy, triggering tissue remodeling, hypoxia, and immune cell infiltration. This environment transforms adipose tissue into a site of low-grade, chronic inflammation—a hallmark of insulin resistance (27, 28).

In obese individuals, there is a marked shift in adipose-resident immune cell populations. Macrophages, which typically comprise ~10% of cells in lean adipose tissue, increase to over 40–50% in obesity. Furthermore, there is a phenotypic switch from anti-inflammatory M2-like macrophages to proinflammatory M1-like macrophages, which produce TNF-α, IL-6, and inducible nitric oxide synthase (iNOS), directly impairing insulin signaling in adipocytes (29–31).

Compounding this, adipocyte hypoxia, driven by rapid adipose expansion outpacing vascular supply, leads to fibrosis and increased chemokine secretion (e.g., MCP-1), promoting monocyte recruitment and further macrophage accumulation (32). These macrophages form crown-like structures around dead or dying adipocytes—a histological hallmark of inflamed adipose tissue.

The proinflammatory milieu increases circulating free fatty acids (FFAs) through enhanced lipolysis (33), which, in turn, activate toll-like receptors (TLRs) and stress signaling pathways in peripheral tissues, exacerbating insulin resistance (**Figure 2B**).

### 2.4 Hypoxia-mediated insulin resistance

Hypoxia plays a central role in adipose tissue dysfunction. As adipocytes expand, oxygen diffusion becomes limited, inducing hypoxia-inducible factor-1 alpha (HIF1α) expression. HIF1α activates transcription of genes involved in glycolysis, angiogenesis, and inflammation. In obese adipose tissue, overexpression of HIF1α has been linked to upregulation of chemokines such as MCP-1 and leukotriene B4 (LTB4), which recruit proinflammatory macrophages (34–36).

Mechanistically, hypoxia activates the JNK and NF-κB pathways, which disrupt insulin signaling by promoting serine phosphorylation of insulin receptor substrate-1 (IRS-1), impairing downstream AKT activation (37–39). These stress pathways also enhance the expression of inducible nitric oxide synthase (iNOS), further compromising vascular function and insulin action.

### 2.5 Dietary fatty acids in insulin resistance

High-fat diets rich in saturated fatty acids (e.g., palmitate) are potent inducers of insulin resistance. Saturated fats activate pattern recognition receptors such as TLR2 and TLR4, triggering inflammatory cascades via the MyD88-dependent NF-κB and JNK pathways. These pathways upregulate inflammatory cytokines, impair insulin receptor signaling, and induce endoplasmic reticulum stress (40, 41).

Moreover, saturated fats activate the NLRP3 inflammasome, leading to IL-1β and IL-18 maturation. These cytokines not only promote insulin resistance but also induce β-cell dysfunction and apoptosis. In contrast, unsaturated fatty acids (e.g., oleate) have anti-inflammatory effects, underscoring the importance of fat quality in metabolic health.

### 2.6 Hypothalamic insulin resistance

Obesity, particularly abdominal obesity, elevates circulating free fatty acids and pro-inflammatory mediators that impair insulin signaling in both peripheral tissues and the central nervous system. Peripheral insulin resistance drives hyperinsulinemia, which can exacerbate central insulin resistance by disrupting hypothalamic leptin signaling and glucose sensing. Central insulin resistance, in turn, compromises hypothalamic regulation of appetite, promoting hyperphagia and further weight gain (42). This interplay establishes a self-reinforcing cycle in which peripheral and central insulin resistance mutually exacerbate one another. Dysregulated appetite arises as a key consequence: hyperinsulinemia may enhance hunger, while impaired central insulin signaling reduces the brain’s responsiveness to satiety cues, collectively contributing to obesity progression and metabolic dysfunction. In rodent models, overnutrition activates the IKKβ/NF-κB signaling axis in hypothalamic neurons, promoting the production of IL-6 and TNF-α, which disrupt insulin receptor signaling (43, 44).

ER stress and JNK activation impair leptin and insulin action, contributing to hyperphagia and weight gain (45–47). Inflammatory adipokines such as *resistin* and FFAs reach the hypothalamus via the circulation and cerebrospinal fluid, triggering TLR4 activation and promoting serine phosphorylation of IRS-1, which blocks downstream PI3K/AKT signaling (48, 49). Management of insulin resistance targets both peripheral and central mechanisms through lifestyle interventions—caloric restriction, balanced diet, and regular physical activity—which improve peripheral insulin sensitivity and enhance central insulin and leptin signaling, complemented by pharmacological agents when lifestyle measures are insufficient.

### 2.7 Complement system activation

The complement system, traditionally known for its role in innate immunity, also plays a pivotal role in adipose tissue biology and insulin resistance. Adipose tissue produces complement components such as *adipsin (Factor D)* and C3, both of which are upregulated in obesity (50–52). Adipsin is required for adipocyte differentiation and survival, while C3-derived products such as C3a and C3adesArg (ASP) influence lipid storage and glucose metabolism (53, 54).

Obese individuals exhibit elevated levels of C3a, which signals through the C3a receptor (C3aR) to promote inflammation and macrophage activation (55). Similarly, C5a and its receptor C5aR mediate immune cell recruitment and cytokine production. While ASP exerts insulin-like effects in lean individuals by promoting triglyceride synthesis, ASP resistance develops in obesity, contributing to dysregulated lipid storage and insulin resistance (56). Dedifferentiation of islet β-cells is increasingly recognized as a central event in the progression of type 2 diabetes mellitus (T2DM) (57). It was observed that complement C3 is elevated in the circulation and islets of T2DM patients and mice, where it drives β-cell dedifferentiation (50). Conversely, treatment with insulin, gliclazide, or metformin lowered C3, Nga3, and Oct4 expression while restoring Pdx1 and MafA, thereby protecting β-cell identity. Mechanistic studies revealed that C3 activates Wnt/β-catenin signaling through phosphorylation of β-catenin, and pathway inhibition effectively blocked C3-induced dedifferentiation. Collectively, these results identify C3 as a key mediator of β-cell dedifferentiation in T2DM and support its inhibition as a potential strategy to preserve β-cell function (58).

## 3 Obesity in the development and progression of CVDs

Obesity significantly increases the risk of cardiovascular diseases (CVDs) through the complex interplay of metabolic, hemodynamic, inflammatory, and neurohormonal mechanisms. Multiple epidemiological studies have established obesity as a major risk factor for hypertension, dyslipidemia, atherosclerosis, coronary artery disease, and stroke (7, 59). One of the central pathological processes linking obesity to CVD is atherosclerosis, which is exacerbated by the chronic low-grade inflammation, oxidative stress, and metabolic dysregulation characteristic of obesity.

**Figure 3:**
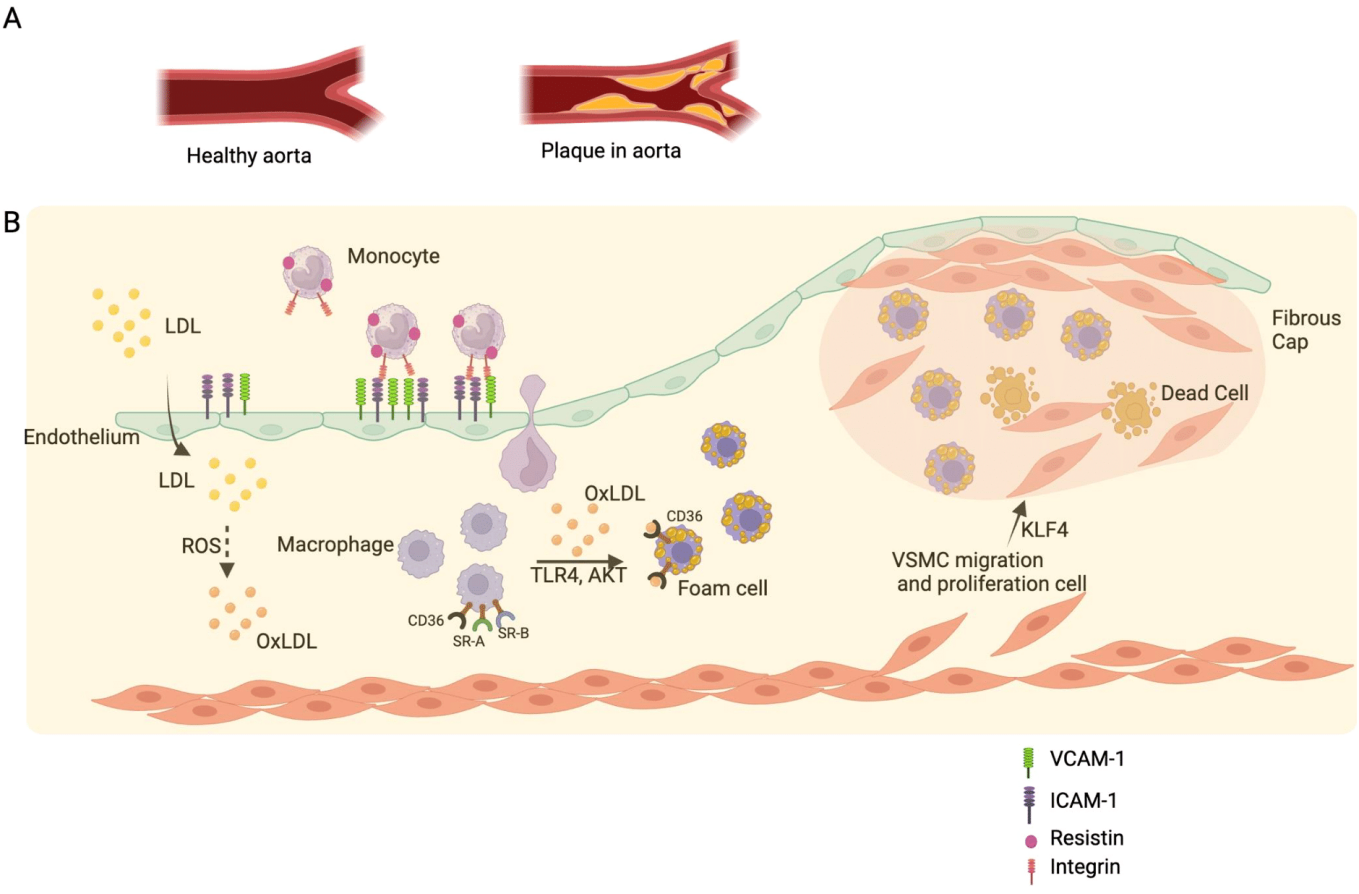
Pathogenesis of atherosclerosis. **(A)** Comparison of a healthy aorta with an aorta affected by atherosclerotic plaque. **(B)** Schematic representation of atherosclerotic plaque formation. Low-density lipoprotein (LDL) particles infiltrate the subendothelial space and become oxidized (OxLDL) due to reactive oxygen species (ROS) produced by endothelial cells. Endothelial cells, activated by OxLDL, express adhesion molecules such as VCAM-1 and ICAM-1, which facilitate the recruitment and adhesion of circulating monocytes. Monocytes differentiate into macrophages and engulf OxLDL via scavenger receptors (SR-A, SR-B, CD36) and through TLR4-mediated signaling pathways. This uptake transforms macrophages into foam cells, which accumulate in the subendothelial space. As the lesion progresses, smooth muscle cells (VSMCs) migrate from the media into the intima and proliferate, influenced by factors like KLF4. The accumulation of foam cells, VSMCs, and extracellular matrix components leads to the formation of a fibrous cap overlying a necrotic core composed of dead cells and lipid debris, characteristic of an advanced atherosclerotic plaque.

In individuals with obesity, adipose tissue—especially visceral fat—acts as an active endocrine organ that secretes various adipokines (e.g., leptin, resistin, adiponectin), pro-inflammatory cytokines (e.g., TNF-α, IL-6), and chemokines (e.g., MCP-1) (60). These factors promote endothelial dysfunction, vascular inflammation, and lipid accumulation within the arterial wall. Endothelial cells exposed to inflammatory signals upregulate adhesion molecules such as VCAM-1 and ICAM-1, which recruit circulating monocytes into the subendothelial space. These monocytes differentiate into macrophages that engulf oxidized low-density lipoprotein (OxLDL) via scavenger receptors such as CD36 and scavenger receptor A (SRA), transforming into foam cells and initiating fatty streak formation—the earliest visible lesion of atherosclerosis (61) (**Figure 3**).

Over time, continued lipid accumulation, cellular apoptosis, and inflammatory activation promote the progression of fatty streaks into fibrous plaques. Leptin and resistin, which are elevated in obesity, further aggravate plaque instability by inducing reactive oxygen species (ROS), smooth muscle cell proliferation, and extracellular matrix remodeling. Leptin also enhances platelet aggregation and thrombosis, while resistin impairs endothelial nitric oxide (NO) production, increasing vasoconstriction and reducing vascular repair capacity (62).

Dyslipidemia in obesity—characterized by elevated triglycerides, increased LDL particles (especially small dense LDL), and reduced high-density lipoprotein (HDL)—contributes further to atherogenesis. Insulin resistance exacerbates this dyslipidemia by increasing hepatic very-low-density lipoprotein (VLDL) production and reducing HDL synthesis.

Thus, obesity accelerates every stage of atherogenesis, from endothelial dysfunction to plaque rupture, ultimately heightening the risk of myocardial infarction, stroke, and peripheral arterial disease. Moreover, obesity-related metabolic and inflammatory factors interfere with normal cardiac and vascular physiology even in the absence of overt atherosclerosis, increasing susceptibility to various forms of cardiovascular pathology.

## 4 Obesity as a pathogenic driver of heart failure

Heart failure (HF) is a major complication of obesity, with pathophysiology extending beyond the contributions of traditional risk factors like hypertension and diabetes. The development of obesity-related HF is a multifactorial process involving hemodynamic overload, structural cardiac remodeling, metabolic derangements, chronic inflammation, and neurohormonal activation (63). In the early stages of obesity, increased blood volume and cardiac output due to expanded adipose tissue mass impose a chronic volume and pressure load on the heart. This hemodynamic stress triggers adaptive left ventricular hypertrophy (LVH) to maintain cardiac output. However, prolonged overload results in maladaptive remodeling, characterized by cardiomyocyte hypertrophy, interstitial fibrosis, and impaired diastolic filling, hallmark features of heart failure with preserved ejection fraction (HFpEF), which is highly prevalent in obese individuals (64).

Chronic low-grade inflammation and oxidative stress, common in obesity, directly contribute to myocardial injury and remodeling. Pro-inflammatory cytokines such as TNF-α and IL-6 activate cardiac fibroblasts, promote extracellular matrix deposition, and reduce myocardial compliance. Insulin resistance induces metabolic inflexibility in the heart, impairing glucose uptake and increasing reliance on fatty acid oxidation. This shift leads to mitochondrial dysfunction and the accumulation of lipotoxic intermediates, which damage cardiomyocytes. Locally, epicardial adipose tissue (EAT), significantly increased in obesity, secretes pro-inflammatory adipokines and cytokines in proximity to the myocardium, exacerbating myocardial fibrosis and coronary microvascular dysfunction.

Neurohormonal systems, including the renin–angiotensin–aldosterone system (RAAS) and the sympathetic nervous system (SNS), are activated in obesity. Elevated leptin levels stimulate sympathetic activity, contributing to increased heart rate, vasoconstriction, and sodium retention, thereby increasing cardiac workload (65). Over time, this metabolic and structural strain can lead to a deterioration of systolic function, resulting in heart failure with reduced ejection fraction (HFrEF). Obesity induces significant structural changes in the heart, collectively called cardiac remodeling. These include left ventricular hypertrophy (LVH), increased left ventricular mass, chamber dilation, and concentric or eccentric remodeling. Echocardiographic and MRI studies consistently show that obese individuals exhibit a higher prevalence of LVH, which initially maintains cardiac output but ultimately compromises diastolic filling (66).

**Figure 4:**
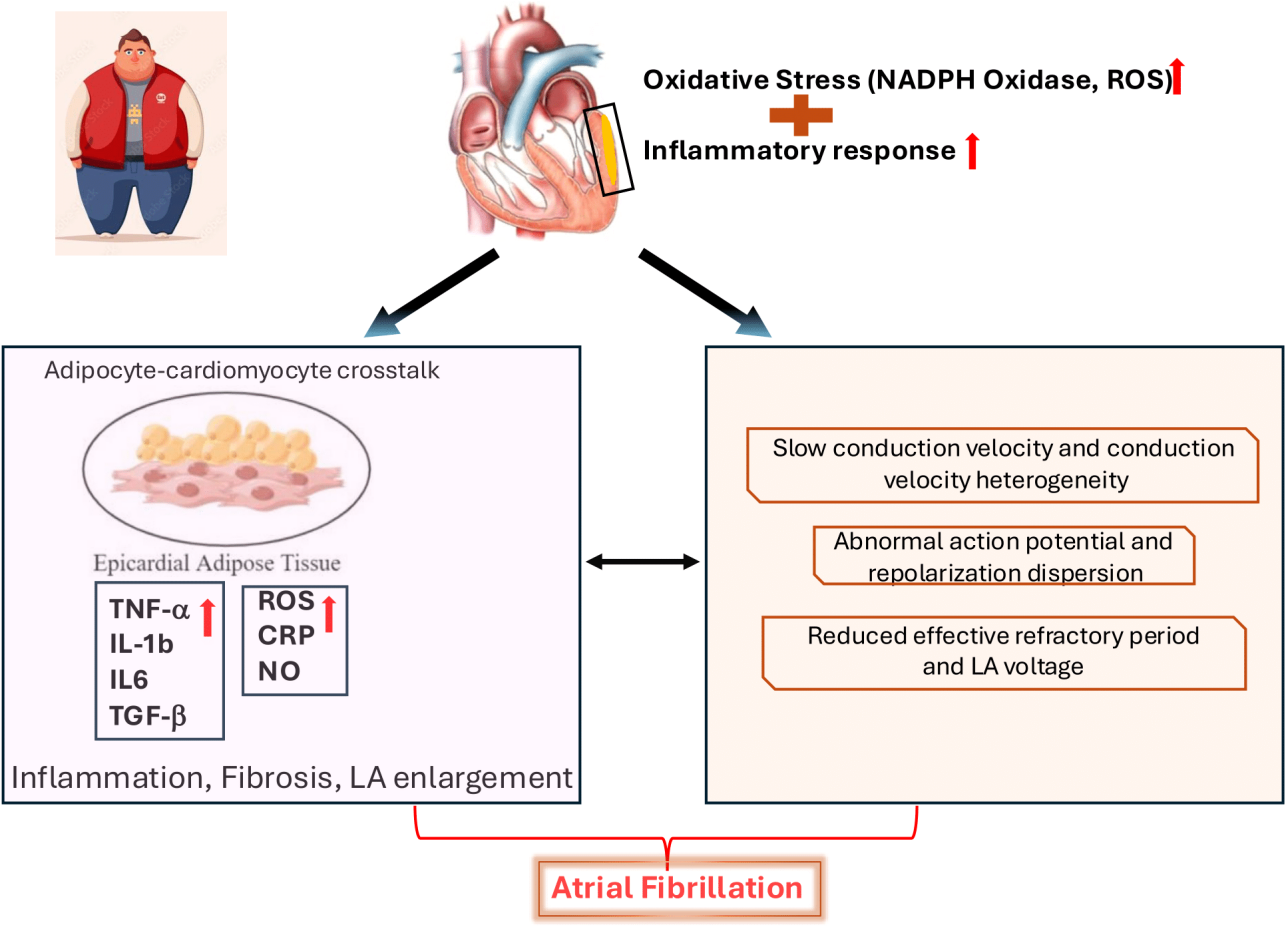
Mechanisms linking obesity and atrial fibrillation. In the heart, obesity is associated with increased oxidative stress, primarily through NADPH oxidase and reactive oxygen species (ROS) production, as well as an elevated inflammatory response. These factors directly contribute to electrophysiological abnormalities in the atria, including slow and heterogeneous conduction velocity, abnormal action potentials, increased repolarization dispersion, reduced effective refractory period, and decreased left atrial (LA) voltage, all of which are pro-arrhythmic. Furthermore, obesity leads to changes in epicardial adipose tissue, fostering “adipocyte-cardiomyocyte crosstalk.” This involves increased secretion of pro-inflammatory cytokines like TNF-α, IL-1β, and IL-6, along with elevated ROS and CRP, while nitric oxide (NO) levels may be altered. This crosstalk promotes inflammation and fibrosis within the atria, leading to LA enlargement. Both the direct cardiac effects (oxidative stress, inflammation, and electrophysiological changes) and the effects mediated by epicardial adipose tissue contribute to the development and maintenance of atrial fibrillation.

Furthermore, obesity significantly elevates the risk of arrhythmias, particularly atrial fibrillation (AF), which contributes to HF morbidity. Arrhythmogenesis is driven by systemic and local myocardial inflammation, oxidative stress, abnormal autonomic tone, and structural changes (67). Leptin and other adipokines can alter cardiomyocyte ion channel expression and electrophysiological properties, prolonging action potential duration and delaying repolarization. Critically, adipose infiltration into the atrial myocardium and interstitial fibrosis create areas of conduction block and re-entry circuits, predisposing to AF. Obesity-related sleep apnea and intermittent hypoxia further exacerbate this arrhythmogenic substrate by increasing sympathetic activity and ROS production (**Figure 4**).

In summary, obesity drives HF through a convergent path of hemodynamic, metabolic, inflammatory, and structural insults that remodel the heart and disrupt its electrophysiological stability.

## 5 Conclusion

This review underscores the intricate relationship between obesity and its associated comorbidities, including type 2 diabetes mellitus (T2DM), atherosclerosis, and cardiomyopathy, highlighting potential underlying mechanisms. Numerous studies have shown that obesity promotes chronic low-grade inflammation across multiple tissues. This inflammatory state is characterized by the accumulation and polarization of both innate and adaptive immune cells toward pro-inflammatory phenotypes. Sustained inflammation contributes significantly to the development of insulin resistance and the progression to T2DM.

In addition to peripheral insulin resistance, obesity impairs insulin sensitivity in the hypothalamus, where the central Resistin/TLR4 signaling axis has been implicated in promoting hypothalamic inflammation and systemic metabolic dysfunction.

Emerging research also emphasizes the multifaceted role of the complement system in obesity-related pathologies. Traditionally recognized for its role in immune surveillance and host defense, the complement system also modulates metabolic inflammation by clearing pathogens and cellular debris. Although it may not serve as a primary driver of disease, the complement system can influence both the initiation and resolution of inflammatory responses, increasingly being recognized as a key contributor to obesity-induced insulin resistance and metabolic dysregulation.

Obesity also heightens the risk of cardiovascular diseases, including hypertension, atherosclerosis, atrial fibrillation, left ventricular remodeling, and heart failure (HF). These risks arise from a complex interplay of metabolic, hemodynamic, and inflammatory mechanisms. Interestingly, despite these risks, multiple studies have observed a lower mortality rate among overweight and obese individuals with pre-existing cardiovascular conditions compared to their leaner counterparts—a phenomenon known as the “obesity paradox.”

Although obesity is a well-established risk factor for cardiovascular disease, diabetes, and overall mortality, multiple epidemiological studies have reported an “obesity paradox,” in which overweight or moderately obese individuals exhibit better survival in certain chronic conditions, including heart failure, coronary artery disease, chronic kidney disease, and type 2 diabetes. Large cohort studies of heart failure and post–myocardial infarction populations consistently show lower all-cause and cardiovascular mortality among overweight and mildly obese patients compared with those of normal weight. However, these observations are largely derived from observational data and may be influenced by biases such as reverse causation, selection bias, and residual confounding, as well as limitations of body mass index (BMI) in capturing fat distribution and lean mass.

Several mechanisms have been proposed to explain this paradox. Excess adiposity may provide metabolic reserves during acute illness or catabolic stress, protecting against tissue breakdown and malnutrition. Fat distribution is also critical: while visceral fat is strongly associated with adverse metabolic and cardiovascular outcomes, subcutaneous fat may exert protective effects by modulating systemic inflammation and maintaining insulin sensitivity. Adipose tissue secretes bioactive hormones, or adipokines, such as adiponectin, which possess anti-inflammatory and cardioprotective properties, potentially moderating disease severity. Furthermore, obese individuals often present earlier in the disease course and may receive more intensive medical care, while preserved skeletal muscle mass and higher cardiorespiratory fitness may enhance resilience and survival (68–70).

Recent studies using more precise measures of body composition, including visceral fat quantification, waist-to-hip ratio, and lean mass assessment, suggest that the apparent protective effects of obesity are largely limited to individuals with greater subcutaneous fat and preserved muscle mass. Collectively, these findings indicate that the obesity paradox arises from a combination of physiological mechanisms, fat distribution and adipokine effects, methodological biases, and limitations of traditional anthropometric measures, underscoring the need for nuanced interpretation in clinical practice (71, 72).

Nevertheless, in most individuals, obesity creates a pro-inflammatory milieu that facilitates monocyte recruitment into the vascular wall. These monocytes differentiate into macrophages, which contribute to foam cell formation by engulfing oxidized LDL, initiating the development of atherosclerotic plaques. As global obesity rates rise, so too does the number of individuals at heightened risk for heart failure. Obesity promotes several metabolic disturbances—such as insulin resistance, dysregulated adipokine production, chronic inflammation, and myocardial lipotoxicity—all of which contribute to cardiac dysfunction.

Furthermore, obesity is a key contributor to secondary conditions that exacerbate HF risk, including obstructive sleep apnea (OSA) and obesity hypoventilation syndrome (OHS). It also drives structural, functional, and electrophysiological changes in the myocardium, including ventricular hypertrophy, fibrosis, and arrhythmias. Understanding these interlinked mechanisms is essential for the prevention and management of HF. Moreover, addressing the paradoxical impact of obesity on heart failure outcomes is crucial for guiding evidence-based weight management strategies tailored to individual patient profiles.

## 6 Future research directions

Future research on obesity must adopt a more holistic and integrative framework that captures its biological complexity and clinical heterogeneity. Rather than focusing solely on BMI as a risk marker, efforts should shift toward understanding how fat distribution, tissue-specific signaling, and systemic inflammatory and endocrine networks interact to shape disease trajectories. Emerging tools—including single-cell and spatial omics, advanced imaging, and systems biology approaches—offer unprecedented opportunities to dissect these pathways in detail and uncover novel therapeutic targets. A deeper exploration of paradoxical observations, such as the protective role of subcutaneous adiposity or the survival advantage seen in certain obese populations, may reveal adaptive mechanisms with broad clinical relevance. Equally important is the integration of mechanistic insights with population-level data to refine risk prediction and guide precision interventions. By embracing this multidimensional approach, the field can move closer to transforming obesity research into actionable strategies that not only prevent and treat comorbidities but also improve long-term health outcomes on a global scale.
